# Supplementary material for: Online parent training platform for complementary treatment of disruptive behavior disorders in attention deficit hyperactivity disorder: A randomized controlled trial protocol
Source: PLoS One. 2022 Oct 27;17(10):e0272516. doi: 10.1371/journal.pone.0272516 (PMC9612579; doi:10.1371/journal.pone.0272516)
Supplement: S2 File — (DOCX) [file pone.0272516.s002.docx]

**Informed consent materials**

**Consent form:**

**Termo de Consentimento Livre e Esclarecido**

Título da Pesquisa: Plataforma Online de Treinamento de Pais para tratamento complementar de Transtornos do Comportamento Disruptivo no Transtorno de Déficit de Atenção/Hiperatividade

Prezado (a),

Gostaríamos de convidá‐lo (a) para participar de uma pesquisa cujo objetivo é investigar a eficácia de um tratamento não‐farmacológico, voltado para diminuição dos sintomas disruptivos e externalizantes de crianças com TDAH. O tratamento consiste no treinamento de manejo comportamental e é realizado com os pais da criança, tendo por objetivo desenvolver estratégias de como lidar com o mau comportamento e se tornar mais assertivo com seu filho (a). Nesta pesquisa estudaremos a viabilidade dessa intervenção em um formato online, para possibilitarmos futuramente um acesso mais amplo à intervenção. Nosso convite é para que participe desta pesquisa no grupo:

( ) 1. Famílias que passarão por intervenção presencial (crianças com TDAH e/ou TOD); ( ) 2. Famílias que passarão por intervenção online (crianças com TDAH e/ou TOD); ( ) 3.Famílias que estarão na fila de espera para a intervenção (grupo controle; crianças com TDAH e/ou TOD).

Leia as informações abaixo antes de autorizar ou não sua participação na pesquisa.

Os transtornos do Comportamento Disruptivo estão entre as condições mais facilmente identificáveis entre aquelas coexistentes com o TDAH, pois envolvem comportamentos notáveis como “crises de birra”, agressão física, argumentação excessiva, roubo e outras formas de desafio e resistência à autoridade. O transtorno disruptivo mais frequentemente coexistente com o TDAH é o Transtorno de Oposição Desafiante, podendo chegar a 50% dos casos. Esses comportamentos disruptivos causam prejuízos significativos a curto e longo prazo. Intervenções efetivas na infância, como o Treinamento de Pais, podem resultar no controle desses comportamentos, e promover uma diminuição significativa desses prejuízos.

Caso você autorize a sua participação e de seu (sua) filho (a) na pesquisa, algumas informações sobre o seu comportamento serão coletadas, além disso informações sobre a história e o comportamento da criança serão solicitadas através do preenchimento de diversos questionários, e através de uma entrevista de aproximadamente 60 minutos de duração. Você e a criança passarão ainda por uma avaliação cognitiva com duração de 20 minutos, aproximadamente. Após essa triagem inicial, você iniciará o Treinamento de Pais, de forma presencial ou online, ou permanecerá em uma fila de espera (caso não haja mais vagas), sendo submetida à intervenção mais tarde.

O treinamento de pais será realizado em 6 sessões/módulos. A versão presencial será realizada em grupo (4 grupos de 5 famílias) e contará, portanto, com 6 sessões guiadas por terapeuta especializado com duração de aproximadamente 60 minutos cada. O TP online, contará com 6 módulos, cujos objetivos serão análogos aos das sessões presenciais. Na versão online, o pai terá acesso irrestrito à conteúdo multimídia como cartilhas informativas, vídeos em formato de animações ou gravações, entre outros materiais, de maneira sequencial e considerando um módulo por semana. Para cada módulo a duração aproximada dos vídeos é de 20 minutos e eles poderão ser assistidos por você mais de uma vez. Ainda nesta versão, o pai deverá responder algumas questões acerca do que foi abordado naquele módulo e deverá ter 60% de aproveitamento para ter acesso ao módulo seguinte, sem contato com nenhum profissional. O tempo total de duração por módulo online é de aproximadamente 30 minutos. Na versão presencial, o terapeuta estará inteiramente disponível para eventuais dúvidas e o material didático disponibilizado será restrito apenas à cartilhas informativas. Uma reavaliação utilizando algumas escalas será feita após a terceira sessão/módulo, e novamente ao final do TP, após a 6ª sessão/módulo. O preenchimento dessas escalas tem duração aproximada de 20 minutos. A triagem inicial e a intervenção presencial serão realizadas no Centro de Tecnologia em Medicina Molecular, na faculdade de medicina da UFMG, por profissional especializado.

O risco envolvido na pesquisa é mínimo. Em relação à avaliação comportamental e cognitiva, o risco máximo é de desconforto físico e ansiedade relacionada aos procedimentos de testagem. Todo esforço será feito no sentido de atentar para o bem‐estar físico e psicológico dos participantes, interrompendo‐se a testagem aos menores sinais de desconforto, além de se adotar procedimentos de relaxamento e esclarecimento. Não existem riscos relacionados à intervenção.

A sua participação e da criança na pesquisa é voluntária. Será ofertado para o participante e seu acompanhante um lanche durante a realização dos procedimentos e o recurso de despesas decorrentes de deslocamento será provido conforme resolução. Você poderá optar, em qualquer momento da pesquisa, pelo encerramento da participação da criança sem sofrer nenhum tipo de prejuízo. Os resultados da pesquisa poderão ser utilizados em trabalhos científicos publicados ou apresentados oralmente em congressos e palestras sem revelar a identidade dos participantes. Ao final, você obterá oralmente e por escrito, sob a forma de aconselhamento e de um relatório, os resultados da análise dos seus dados e de seu (sua) filho (a) realizada por profissionais das áreas da pediatria e psicologia. Caso seja identificado algum problema de saúde ou alguma necessidade educacional, a família será orientada e a criança encaminhada para os serviços disponíveis na comunidade com o objetivo de otimizar sua saúde, bem‐ estar e desempenho funcional.

Agradecemos sua atenção e valiosa colaboração.

Atenciosamente,

Prof. Dr. Marco Aurélio Romano Silva ‐ CRM‐23889 Prof. Titular do Departamento de Saúde Mental da UFMG

Av. Prof. Alfredo Balena, 190 ‐ sala 114 / Belo Horizonte – MG; Telefone: (31) 3409 9650

Para maiores esclarecimentos ou em caso de dúvidas sobre ética: Comitê de Ética em Pesquisa (COEP‐UFMG), na Av. Antônio Carlos, 6627 – Unidade administrativa II, 2º andar/ Campus Pampulha‐ UFMG. Tel: (31)34094592/ E‐mail: [coep@prpq.ufmg.br](mailto:coep@prpq.ufmg.br)

Responsável

Eu, , abaixo assinado (a), declaro ter sido informado (a) sobre os procedimentos e propostas da pesquisa ‘Plataforma Online de Treinamento de Pais para tratamento complementar de Transtornos do Comportamento Disruptivo no Transtorno de Déficit de Atenção/Hiperatividade’ e concordo com a participação voluntária da criança , pela qual sou responsável.

Belo Horizonte, de de .

Assinatura

Participante

Eu, ,abaixo assinado (a), declaro ter sido informado (a) sobre os procedimentos e propostas da pesquisa ‘Plataforma Online de Treinamento de Pais para tratamento complementar de Transtornos do Comportamento Disruptivo no Transtorno de Déficit de Atenção/Hiperatividade ’ e concordo com a minha participação voluntária.

Belo Horizonte, de de .

Assinatura

**Assent form:**

**Termo de Assentimento**

Olá!

Este é um convite para que você participe como voluntário de uma pesquisa chamada “Plataforma Online de Treinamento de Pais para tratamento complementar de Transtornos do Comportamento Disruptivo no Transtorno de Déficit de Atenção/Hiperatividade”. Nela nós queremos saber se um tratamento feito pela internet funciona. Esse tratamento serve para os pais aprenderem como diminuir alguns maus comportamentos nos filhos (como pirraça ou agressividade). Esses problemas podem acontecer quando a criança é muito desatenta, agitada e se costuma ter dificuldade em seguir regras e obedecer seus pais ou professores. Esse tratamento é como um treinamento e vai ser feito só com os seus pais, ensinando pra eles como conversar de um jeito melhor com você, como te ajudar a se comportar bem, como prestar mais atenção às coisas legais que você faz e como ser firmes quando necessário. Nesta pesquisa estudaremos se esse treinamento pode funcionar quando é feito pela internet, dessa forma, muitas famílias futuramente poderão ter acesso à ele e assim a relação entre muitas crianças e seus pais poderá ser mais saudável!

Nessa pesquisa, você vai vir para uma consulta e vamos conversar com você antes de começarmos com os seus pais. Nesse encontro, faremos um jogo, que envolve raciocínio lógico! O jogo serve para avaliarmos sua inteligência. Além disso, seus pais vão passar por uma entrevista e preencher diversas informações a respeito do seu comportamento. Terminada essa primeira etapa, iniciaremos com os seus pais. O treinamento com eles durará 6 semanas, lembrando que o objetivo é melhorar a relação de vocês! Não quer participar? Não tem problema, você tem todo o direito de negar. Mesmo após começar, você pode interromper a qualquer momento, pois não é obrigado a ir até o fim. Mesmo depois do nosso encontro você pode desistir e não incluiremos você e sua família na pesquisa. Se você aceitar participar, você e o seu pai/mãe ou responsável deverão autorizar, assinando um convite como este.

Você terá riscos ao participar? Não, pois você participará apenas de um jogo que envolve perguntas e respostas. No entanto, algumas pessoas ficam entediadas durante essas tarefas. Caso isso aconteça, podemos fazer uma pausa para descanso ou até mesmo parar o jogo. A equipe estará pronta para te ajudar em tudo o que for necessário e relacionado à pesquisa. Em relação aos encontros com seus pais, há uma grande chance que eles tragam benefícios para vocês.

Sobre os resultados, ninguém, tirando os pesquisadores, terão acesso a eles. Você e seus pais também podem ter acesso, e para isso basta entrar em contato conosco. Por fim, o resultado dessa pesquisa será apresentado em palestras, aulas e será escrito para que outros pesquisadores leiam e utilizem em suas pesquisas e atendimentos. Mas não se preocupe, o seu nome não aparecerá de jeito nenhum. Só serão divulgados os dados de todo mundo junto e ninguém saberá que você participou da pesquisa. Após a análise dos questionários e testes, eles serão arquivados sob a responsabilidade da pesquisadora Débora Marques de Miranda ([debora.m.miranda@gmail.com](mailto:debora.m.miranda@gmail.com)).

Qualquer dúvida sobre a pesquisa pode ser tirada com a pesquisadora responsável pelo e‐mail [nitida.hc.ufmg@gmail.com](mailto:nitida.hc.ufmg@gmail.com) ou pessoalmente no Instituto Nacional de Ciência e Tecnologia – Medicina Molecular, com unidade localizada na Faculdade de Medicina da UFMG (Av. Alfredo Balena, 190, sala 114, Santa Efigênia,Belo Horizonte ‐ MG).

Este termo será emitido em duas cópias iguais. Uma fica com você e outra com a pesquisadora. Caso tenha dúvidas sobre questões éticas, entre em contato com o Comitê de Ética em Pesquisa – COEP/UFMG ao qual este projeto foi submetido: Avenida Presidente Antônio Carlos 6627, Unidade Administrativa II – 2º andar, sala 2005. CEP 31270901. BH‐MG. Telefone: (031) 3409‐4592.

Muito obrigado por sua atenção e valiosa colaboração!

Professora Dra. Débora Marques de Miranda Fac. de Medicina da Universidade Federal de Minas Gerais Av. Prof. Alfredo Balena, 190, sl. 114/Belo Horizonte – MG

Eu, , aceito participar da pesquisa “Plataforma Online de Treinamento de Pais para tratamento complementar de Transtornos do Comportamento Disruptivo no Transtorno de Déficit de Atenção/Hiperatividade”. Declaro que li o termo de assentimento junto com o pesquisador e entendi o que é a pesquisa e para que ela serve. Sei também que os meus resultados serão mantidos em absoluto sigilo e que serão utilizados apenas para esta pesquisa.

Belo Horizonte, de de .

Assinatura
