# Supplementary material for: Online parent training platform for complementary treatment of disruptive behavior disorders in attention deficit hyperactivity disorder: A randomized controlled trial protocol
Source: PLoS One. 2022 Oct 27;17(10):e0272516. doi: 10.1371/journal.pone.0272516 (PMC9612579; doi:10.1371/journal.pone.0272516)
Supplement: S3 File — (DOCX) [file pone.0272516.s003.docx]

**Administrative information**

**Title {1}**

Online Parent Training Platform for complementary treatment of Disruptive Behavior Disorders in Attention Deficit Hyperactivity Disorder: a randomized controlled trial protocol

**Trial registration**

**Registry {2a}**

Registered on Brazilian Registry of Clinical Trials (ReBEC). Register Number: RBR-6cvc85.

**Data Set {2b}**

Date of registration: July 24^th^ (2020) 05:35 pm.

Date recruitment began: 25/05/2021; Approximate date when recruitment will be completed: 25/05/2022.

**Protocol version {3}**

July 24^th^ (2020). First version.

**Roles and Responsibilities** **{5}**

**Contributorship {5a}**

Gabrielle Chequer de Castro Paiva. Master and PhD student in Molecular Medicine (Federal University of Minas Gerais - UFMG).

Daniel Augusto Ferreira and Santos. Master in Molecular Medicine (UFMG).

Julia Silva Jales. Psychologist (UFMG). Volunteer member of Research Center of Impulsivity and Attention.

Marco Aurélio Romano-Silva. Doctor in Biochemistry (UFMG). Full Professor, Department of Mental Health, Faculty of Medicine, UFMG.

Débora Marques de Miranda. Master and Doctor in Biochemical and Molecular Pharmacology (UFMG). Associate Professor of Pediatrics, Faculty of Medicine, UFMG.

GP is the Chief Investigator; she conceived the study, led the proposal and protocol development. DM, JJ and MR contributed to study design and to development of the proposal and methodology. DA developed the online platform. All authors read and approved the final manuscript.

**Sponsor contact information {5b}**

Not applicable

**Committees {5d}**

**Principal Investigator and Research Psychologist**

Design and conduct of Parent Training: Gabrielle Paiva.

Preparation of protocol and revisions: Gabrielle Paiva and Débora Miranda. Organizing steering committee meetings: Gabrielle Paiva and Débora Miranda.

Publication of study reports: Gabrielle Paiva and Débora Miranda.

**Steering committee**

The composition of the trial committee includes all authors.

Agreement of final protocol: Débora Miranda, Gabrielle Paiva and Daniel Santos.

All lead investigators will be steering committee members.

Recruitment of patients and leasing with principle [sic] investigator: Júlia Jales.

Reviewing progress of study and if necessary, agreeing changes to the protocol and/or investigators brochure to facilitate the smooth running of the study: Débora Miranda.

**Trial Management Committee (TMC)**

Study planning; Organization of steering committee meetings: Gabrielle Paiva and Débora Miranda. Randomization and Data verification: Daniel Santos.

**Data Manager**

Maintenance of trial IT system and data entry; Data verification: Daniel Santos and Júlia Jales.

**Lead Investigators**

Gabrielle Paiva and Débora Miranda
