# Supplementary material for: Online parent training platform for complementary treatment of disruptive behavior disorders in attention deficit hyperactivity disorder: A randomized controlled trial protocol
Source: PLoS One. 2022 Oct 27;17(10):e0272516. doi: 10.1371/journal.pone.0272516 (PMC9612579; doi:10.1371/journal.pone.0272516)
Supplement: S1 Protocol — (PDF) [file pone.0272516.s004.pdf]

Plataforma Online de Treinamento de Pais para tratamento complementar de Transtornos do Comportamento Disruptivo no Transtorno de Déficit de Atenção e Hiperatividade

Tipo do estudo:

Intervenções

Título científico:

-BR	EN
Plataforma Online de Treinamento de Pais para tratamento complementar de Transtornos do Comportamento Disruptivo no Transtorno de Déficit de Atenção e Hiperatividade	Online Parent Training Platform for complementary treatment of Disruptive Behavior Disorders in Attention Deficit Hyperactivity Disorder

Identificação do ensaio

úmero do UT : U1111-1255-9795

Título público:

-BR	EN
Plataforma Online de Treinamento de Pais para tratamento complementar de transtornos comportamentais no Transtorno de Déficit de Atenção e Hiperatividade	Online Parent Training Platform for complementary treatment of behavioral disorders in Attention Deficit Hyperactivity Disorder

Acrônimo científico:

Acrônimo público:

Identificadores secundários:

98623218.2.0000.5149
Órgão emissor: Plataforma Brasil

3.350.743
Órgão emissor: Comitê de Ética em Pesquisa da Universidade Federal de Minas Gerais

Patrocinadores

Patrocinador primário: Faculdade de Medicina da Universidade Federal de Minas Gerais

Patrocinadores secundários:

Instituição: Faculdade de Medicina da Universidade Federal de Minas Gerais

Fontes de apoio financeiro ou material:

Instituição: Coordenação de Aperfeiçoamento de Pessoal de Nível Superior

Instituição: Fundação de Amparo à Pesquisa do Estado de Minas Gerais

Instituição: Secretaria de Estado de Saúde de Minas Gerais

Instituição: Ministério da Saúde

Condições de saúde

Condições de saúde ou problemas:

PT-BR	EN
Distúrbios da atividade e da atenção, Transtorno hipercinético de conduta, Distúrbio desafiador e de oposição	Disturbance of activity and attention, Hyperkinetic conduct disorder, Oppositional defiant disorder

Descritores gerais para as condições de saúde:

PT-BR	EN
**F00-F99:** V - Transtornos mentais e comportamentais	**F00-F99:** V - Mental, behavioural disorders

Descritores específicos para as condições de saúde:

PT-BR	ES	EN
**F90.0:** Distúrbios da atividade e da atenção	**F90.0:** Perturbación de la actividad y de la atención	**F90.0:** Disturbance of activity and attention
**F90.1:** Transtorno hipercinético de conduta	**F90.1:** Trastorno hipercinético de la conducta	**F90.1:** Hyperkinetic conduct disorder
**F91.3:** Distúrbio desafiador e de oposição	**F91.3:** Trastorno opositor desafiante	**F91.3:** Oppositional defiant disorder

Intervenções

Categorias das intervenções

Behavioural

Intervenções:

PT-BR	EN
GRUPO 01: 30 participantes. Tratamento convencional, sem tratamento	GROUP 01: 30 participants. Conventional treatment, without complementary

1 nova mensagem

complementar. Consultas bimensais com médico psiquiatra da infância e adolescência, incluindo tratamento medicamentoso se houver indicação clínica (a critério do médico, de acordo ao protocolo clínico), e sem outro tipo de tratamento complementar.

GRUPO 02: 30 participantes. Tratamento convencional e intervenção comportamental em formato presencial. Consultas bimensais com médico psiquiatra da infância e adolescência, incluindo tratamento medicamentoso se houver indicação clínica (a critério do médico, de acordo ao protocolo clínico), e intervenção comportamental complementar: treinamento de manejo comportamental para pais, em formato presencial, com terapeuta especializado, em seis sessões realizadas em regime semanal, adaptadas do manual “Parent Manegement Training” desenvolvido por Alan Kazdin (2005).

GRUPO 03: 30 participantes. Tratamento convencional e intervenção comportamental em formato online. Consultas bimensais com médico psiquiatra da infância e adolescência, incluindo tratamento medicamentoso se houver indicação clínica (a critério do médico, de acordo ao protocolo clínico), e intervenção comportamental complementar: treinamento de manejo comportamental para pais, em formato online, em uma plataforma desenvolvida para o estudo, em seis módulos, a serem realizados em regime semanal, adaptados do manual “Parent Manegement Training” desenvolvido por Alan Kazdin (2005)

treatment. Bi-monthly consultations with a child and adolescent psychiatrist, including drug treatment if there is a clinical indication (at medical discretion, according to the clinical protocol), and without any other type of complementary treatment.

GROUP 02: 30 participants. Conventional treatment and behavioral intervention in face-to-face format. Bi-monthly consultations with a child and adolescent psychiatrist, including drug treatment if there is a clinical indication (at medical discretion, according to the clinical protocol), and complementary behavioral intervention: parent management training, in face-to-face format, with a specialized therapist, in six sessions held on a weekly basis, adapted from the “Parent Manegement Training” manual developed by Alan Kazdin (2005).

GROUP 03: 30 participants. Conventional treatment and behavioral intervention in online format. Bi-monthly consultations with a child and adolescent psychiatrist, including drug treatment if there is a clinical indication (at medical discretion, according to the clinical protocol), and complementary behavioral intervention: parent management training, in online format, on a platform developed for the study, in six modules, to be carried out on a weekly basis, adapted from the “Parent Manegement Training” manual developed by Alan Kazdin (2005)

Descritores para as intervenções:

PT-BR
F04.754.137.131: Análise do Comportamento Aplicada

ES
F04.754.137.131: Análisis Aplicado de la Conducta

EN
F04.754.137.131: Applied Behavior Analysis

Recrutamento

Situação de recrutamento: Not yet recruiting

País de recrutamento

Brazil

Data prevista do primeiro recrutamento: 2021-01-01

Data prevista do último recrutamento: 2021-06-30

Tamanho da amostra alvo:	Gênero para inclusão:	Idade mínima para inclusão:	Idade máxima para inclusão:
90	M	6 Y	12 Y

Critérios de inclusão:

PT-BR

Famílias cujas crianças tenham Transtorno de Déficit de Atenção/Hiperatividade e/ou Transtorno de Oposição Desafiante; que obrigatoriamente tenham sintomas externalizantes como hiperatividade e/ou desafio; que tenham entre 6 e 12 anos; gênero masculino

EN

Families whose children have Attention Deficit/Hyperactivity Disorder and/or Oppositional Defiant Disorder; children must have externalizing symptoms such as hyperactivity and/or oppositional behavior; children who are between 6 and 12 years old; male gender

Critérios de exclusão:

PT-BR

Famílias cujas crianças tenham comorbidades psiquiátricas graves (i.e. psicoses, transtorno afetivo bipolar, depressão grave), genéticas ou neurológicas; cuja inteligência geral seja inferior ao percentil 5; gênero feminino. Famílias cujos cuidadores principais tenham inteligência geral inferior ao percentil 5; condições psiquiátricas graves diagnosticadas (i.e. psicoses, transtorno afetivo bipolar, depressão grave); e famílias com adversidade social grave no contexto domiciliar (i.e. violência doméstica, abuso físico, pobreza extrema, fome)

EN

Families whose children have severe psychiatric comorbidities (i.e. psychosis, bipolar affective disorder, severe depression), genetic or neurological; whose intelligence is below the 5th percentile; feminine gender. Families whose primary caregivers have intelligence below the 5th percentile or have been diagnosed with serious psychiatric conditions (i.e. psychoses, bipolar affective disorder, severe depression); and families with severe social adversity in the home context (i.e. domestic violence, physical abuse, extreme poverty, hunger)

Tipo do estudo

Desenho do estudo:

PT-BR

Ensaio clínico de tratamento, paralelo, com três braços, aberto, randomizado-controlado

EN

Treatment clinical trial, parallel, with three arms, open, randomized-controlled

Programa de acesso expandido	Enfoque do estudo	Desenho da intervenção	Número de braços	Tipo de mascaramento	Tipo de alocação	Fase do estudo

None	Treatment	Parallel	3	Open	Randomized-controlled	N/A

Desfechos

Desfechos primários:

PT-BR

Mudança no quadro sintomatológico do Transtorno de Déficit de Atenção/Hiperatividade e/ou do Transtorno de Oposição Desafiante, com diminuição nos sintomas externailzantes, determinados pela escala MTA-SNAP-IV: que avalia sintomas do transtorno do déficit de atenção/hiperatividade e transtorno de oposição desafiante em crianças e adolescentes (Mattos, et al., 2006); e pela entrevista K-SADS-PL 2013: entrevista semiestruturada que levanta informações importantes a respeito da história de transtornos psiquiátricos, no presente momento e ao longo da vida e avalia a gravidade da sintomatologia (Caye, et al., 2017)

EN

Change in the symptoms of Attention Deficit/Hyperactivity Disorder and/or Oppositional Defiant Disorder, with a decrease in externalizing symptoms, determined by the MTA-SNAP-IV scale: which assesses symptoms of attention deficit/hyperactivity disorder and Oppositional Defiant Disorder in children and adolescents (Mattos, et al., 2006); and the K-SADS-PL 2013 interview: semi-structured interview that raises important information about the history of psychiatric disorders, at the present time and throughout life and assesses the severity of symptoms (Caye, et al., 2017)

Desfechos secundários:

PT-BR

Mudança no estilo parental, com tendência ao estilo democrático, determinado através do Questionário de Estilos e Dimensões Parentais - QEDP (versão 42 itens): Questionário que avalia como os pais manejam sua relação com os filhos e os comportamentos da criança, considerando as dimensões controle e afeto, sendo baseado em um modelo teórico que categoriza os estilos parentais em quatro possíveis: democrático, autoritário, permissivo e negligente (Oliveira, et al., 2018)

EN

Change in parental style, with a tendency to democratic style, determined through the Parenting Styles and Dimensions Questionnaire - PSDQ (42 items version): Questionnaire that assesses how parents manage their relationship with their children and the child's behavior, considering the control dimensions and affection, being based on a theoretical model that categorizes parenting styles into four possible ones: democratic, authoritarian, permissive and negligent (Oliveira, et al., 2018)

PT-BR

Diminuição no nível de estresse percebido pelos cuidadores, determinado pela Escala de Estresse Percebido: mensura o estresse percebido, ou seja, o quanto os indivíduos percebem as situações como estressantes. É uma escala do tipo likert e contém 14 questões (Cohen, et al., 1983)

EN

Decrease in the level of perceived stress by caregivers, determined by the Perceived Stress Scale: measures perceived stress, that is, how much individuals perceive situations as stressful. It is a likert-type scale and contains 14 questions (Cohen, et al., 1983)

PT-BR	EN
Melhora na qualidade de vida dos pais e das crianças, determinada através de duas escalas: WHOQOL-BREF: avalia qualidade de vida. Instrumento desenvolvido pela organização mundial da saúde, em versão reduzida de 100 para 26 itens (WORLD HEALTH ORGANIZATION, 1996) e Kidscreen-52 - Versão relato dos cuidadores e autorrelato: Avalia a qualidade de vida de crianças e adolescentes (Guedes & Guedes, 2011)	Improvement in the quality of life of parents and children, determined through two scales: WHOQOL-BREF: assesses quality of life. Instrument developed by the World Health Organization, in a version reduced from 100 to 26 items (WORLD HEALTH ORGANIZATION, 1996) and Kidscreen-52 - Version of caregivers' report and self-report: Evaluates the quality of life of children and adolescents (Guedes & Guedes, 2011)

Contatos

Contatos para questões públicas

Nome completo: Débora Marques de Miranda

Endereço: Av. Prof. Alfredo Balena, 190 - Santa Efigênia

Cidade: Belo Horizonte / Brazil

CEP: 30130-100

Fone: +55 31 3409-9753

E-mail: debora.m.miranda@gmail.com

Filiação: Faculdade de Medicina da Universidade Federal de Minas Gerais

Contatos para questões científicas

Nome completo: Débora Marques de Miranda

Endereço: Av. Prof. Alfredo Balena, 190 - Santa Efigênia

Cidade: Belo Horizonte / Brazil

CEP: 30130-100

Fone: +55 31 3409-9753

E-mail: debora.m.miranda@gmail.com

Filiação: Faculdade de Medicina da Universidade Federal de Minas Gerais

Nome completo: Gabrielle Chequer de Castro Paiva

Endereço: Av. Prof. Alfredo Balena, 190 - Santa Efigênia

Cidade: Belo Horizonte / Brazil

CEP: 30130-100

Fone: +55 31 3409-9753

E-mail: gabriellechequer@gmail.com

Filiação: Faculdade de Medicina da Universidade Federal de Minas Gerais

Contatos para informação sobre os centros de pesquisa

Nome completo: Débora Marques de Miranda

Endereço: Av. Prof. Alfredo Balena, 190 - Santa

Efigênia

Cidade: Belo Horizonte / Brazil

CEP: 30130-100

Fone: +55 31 3409-9753

E-mail: debora.m.miranda@gmail.com

Filiação: Faculdade de Medicina da Universidade Federal de Minas Gerais

Anexos

[Público]

http://www.ensaiosclinicos.gov.br/static/attachments/pb_parecer_consubstanciado_cep_3350743.pdf (Parecer Consubstanciado de aprovação do estudo por comitê de ética em pesquisa com registro na Plataforma Brasil)
