# Supplementary material for: Online parent training platform for complementary treatment of disruptive behavior disorders in attention deficit hyperactivity disorder: A randomized controlled trial protocol
Source: PLoS One. 2022 Oct 27;17(10):e0272516. doi: 10.1371/journal.pone.0272516 (PMC9612579; doi:10.1371/journal.pone.0272516)
Supplement: Attachment — Submitted filename: Response to Reviewers.docx [file pone.0272516.s005.docx]

Dear Editor and Reviewers,

Many thanks for your careful revision.

Regarding journal requirements, we have re-reviewed the list of references and PLOS ONE's style requirements. About repository information for our data, the current manuscript does not have preliminary data. The results of the study will be published once the research is completed and deidentified data will be made to be available under reasonable request to the correspondent author. We added this information to the cover letter.

Below are answers to the questions raised by Reviewer #2

**Why did you choose male gender only?**

In order to have a representative sample of the studied population, since the prevalence is much higher in boys and our number of participants is relatively small. Also, the clinical presentation of ADHD in girls can be very different (Hinshaw, 2018).

Hinshaw SP. Attention Deficit Hyperactivity Disorder (ADHD): Controversy, Developmental Mechanisms, and Multiple Levels of Analysis. Annu Rev Clin Psychol. 2018 May 7;14:291-316. doi: 10.1146/annurev-clinpsy-050817-084917. Epub 2017 Dec 8. PMID: 29220204.

**2)Would it be possible for more than one caregiver of the same patient to join the study?**

Yes, it is even recommended that all primary caregivers participate (Kazdin,2005). In the pre-intervention evaluation, there is a question that seeks to investigate the caregivers' intention to participate and in the post-intervention evaluation, a question that seeks to quantify the size of the actual participation of each caregiver.

A change has been made to the text with the intention of clarifying this issue. The change can be found in last paragraph of the "Eligibility criteria" session:

“If there is more than one primary caregiver, it is recommended that everyone participate in the intervention. The effective participation of caregivers will be reported and analyzed along with the results.”

**3)The possibility of watching online videos more than once might be a potential bias since patients in the face-to-face group won't have the same option to revisit the material.**

A fundamental difference from the face-to-face group is the frequent contact with the therapist, who helps parents in the application and not only in the exposure of the content, so we understand that each of the groups has specific favorable aspects inherent to the delivery format of intervention. This can be considered an important strategy to transform a face-to-face intervention into a fully self-directed model. This change must necessarily have materials and strategies to facilitate learning (Breitenstein et al., 2014).

Breitenstein, S. M., Gross, D., & Christophersen, R. (2014). Digital delivery methods of parenting training interventions: a systematic review. Worldviews on Evidence-Based Nursing / Sigma Theta Tau International, Honor Society of Nursing, 11(3), 168–176.

**4) Why didn't you do the post-session knowledge assessment to both face to face and online groups?**

We understand that the evaluation after each session in the face-to-face group was not necessary since the feedback of the learned content is immediate during the session, including the therapist's considerations and interventions to verify that the content is clear and that the participant has no doubts about its application to the case. The idea of the test after the online modules would be precisely to fill the information gap and to rise potential frails.

**5) Will the effect of pharmacotherapy regarding dosing and type of medications be controlled during randomization of patients as it might be a major confounding factor regarding the patient outcomes.**

Pharmacological treatment will be conducted by the medical team, following standard protocol and concomitantly with parent training. Frequency of drug treatment and medium dose will be described along with the results and specific statistical analyzes can be performed according to the results. The intent of the study is to verify the effectiveness of parent training as a complementary treatment to standard care.
